# A Topical Desiccant Agent in Association with Manual Debridement in the Initial Treatment of Peri-Implant Mucositis: A Clinical and Microbiological Pilot Study

**DOI:** 10.3390/antibiotics8020082

**Published:** 2019-06-18

**Authors:** Giorgio Lombardo, Annarita Signoriello, Giovanni Corrocher, Caterina Signoretto, Gloria Burlacchini, Alessia Pardo, Pier Francesco Nocini

**Affiliations:** 1 Dentistry and Maxillo-facial Surgery Section, Department of Surgery, Dentistry, Paediatrics and Gynaecology (DIPSCOMI), University of Verona, Piazzale L.A. Scuro 10, 37134 Verona, Italy; annarita.signoriello@univr.it (A.S.); giovanni.corrocher@univr.it (G.C.); alessia.pardo@univr.it (A.P.); pierfrancesco.nocini@univr.it (P.F.N.); 2 Microbiology Section, Department of Diagnostics and Public Health, University of Verona, Piazzale L.A. Scuro 10, 37134 Verona, Italy; caterina.signoretto@univr.it (C.S.); gloria.burlacchini@univr.it (G.B.)

**Keywords:** peri-implant mucositis, manual debridement, desiccant agent, disinfectant gel, soft tissues, microbial sampling

## Abstract

In patients presenting mucositis, effective sub-gingival debridement is crucial to prevent peri-implantitis. The aim of this randomized study was to assess the three-month (T1) effects of a locally delivered liquid desiccant agent with molecular hygroscopic properties, in association with manual debridement, at sites with peri-implant mucositis. Twenty-three patients presenting at least one implant with no radiographically detectable bone loss, a pocket probing depth (PPD) ≥ 4 mm, and bleeding on probing (BOP), were included. At baseline (T0), patients were randomly assigned to receive the aforementioned desiccant agent before debridement (Test-Group), or a Chlorhexidine 1% disinfectant gel after debridement (Control-Group). Treatments were repeated after seven and 14 days. Peri-implant soft tissue assessment [PPD, BOP, Modified Bleeding Index (mBI), Visible Plaque Index (VPI), and Modified Plaque Index (mPLI)] and microbial sampling were performed at T0 and T1. At T1 the Test-Group presented significantly greater reductions for BOP, mBI, VPI, and mPLI. Concerning the deepest sites of the treated implants, both groups showed statistically significant reductions for BOP and mBI between T0 and T1. Furthermore, the Test-Group exhibited a significant decrease in anaerobic bacteria. Despite these valid outcomes, a complete resolution of the inflammatory conditions was not achieved by any of the groups.

## 1. Introduction

Over the past decades, the utilization of dental implants for oral rehabilitation has achieved predictable outcomes and high success rates [1]. 

The main long-term causes of dental implants failure involve the onset of biological complications, such as mucositis and peri-implantitis, at the peri-implant soft tissue level. While mucositis is defined as the presence of a reversible inflammatory soft tissue infiltrate, peri-implantitis implicates bone loss beyond the physiological crestal remodeling [2,3]. Peri-implant mucositis and peri-implantitis represent an emerging global burden of disease [4,5,6,7]. 

Bacterial biofilm plays a fundamental role in the occurrence of periodontal and peri-implant diseases. It has been assumed that peri-implant mucositis is the precursor of peri-implantitis, as gingivitis for periodontitis. On this basis, the most efficient proposed prevention of peri-implantitis development is the early resolution of pre-existing peri-implant mucositis [7,8]; its treatment goal consists in the eradication or significant reduction of the levels of pathogenic microorganisms, in order to allow proper soft tissue healing.

The effectiveness of scaling and root planing (SRP) alone has been demonstrated for the management of peri-implant mucositis, and valid clinical improvements can be obtained through mechanical, ultrasonic, or laser debridement [9,10]. However, traditional non-surgical approaches have been proved to be efficiently supported by adjunctive therapies, such as antiseptic mouth-rinses, topical application of chlorhexidine, and locally delivered antibiotics [11,12,13,14]. Adjunctive systemic antibiotic administration showed good results [15], even if prudence has to be considered to avoid the emergence of bacterial resistance [16]. 

Nevertheless, the achievement of a healthy and completely inflammation-free peri-implant soft tissue condition is not an easily attainable therapeutic task [17,18].

New antiseptic agents have recently been introduced to improve the supra and sub-gingival debridement efficacy. Among them, a new topical desiccant agent with antiseptic properties has been proposed as an adjunct to mechanical debridement in the non-surgical treatment of chronic periodontitis [19,20,21,22] and the surgical regenerative treatment of peri-implantitis [23]. This desiccant product consists of a liquid solution, which contains a concentrated blend of sulfonic and sulfuric acids compounds. Sulfonated aromatics and free sulfates show a strong affinity to bind to the water present in the biofilm matrix and to quickly detach it, allowing easier destruction and eradication of the biofilm. 

To the best of our knowledge, there are no previous investigations on the potentiality of this desiccant agent in the non-surgical treatment of peri-implant mucositis. In the light of these considerations, the aim of this pilot study was to assess the three-month clinical and microbiological effects of the desiccant solution compared to chlorhexidine, locally delivered in peri-implant sites affected by mucositis, as an adjunct to supra and sub-gingival manual debridement.

## 2. Results

### 2.1. Demographics

Demographics were assessed at baseline. Twenty-three participants attended the study; 12 patients in the Test-Group received a desiccant liquid with molecular hygroscopic properties (*HYBENX^®^ Oral Tissue decontaminant™*, HBX), and 11 patients in the Control-Group received a disinfectant gel (*Chlorhexidine Digluconate Corsodyl™ Dental Gel 1%*, CHX). Patients’ mean age was 58.97 ± 10.09 years.

The overall examined implants were 52, 27 in the Test-Group, and 25 in the Control-Group. The total number of evaluated peri-implant sites was 312, 162 in Test-Group and 150 in the Control-Group. At T0, no significant differences between groups were found for age, sex, smoking habit, ASA status (American Society of Anesthesiologists Classification), TPS (Periodontal Supportive Therapy) sessions/year, and oral home care procedures (Table 1).

### 2.2. Clinical Outcomes

Soft tissues comparison between groups is presented in Table 2. 

At baseline, no statistical differences between groups were found for overall peri-implant plaque accumulation and soft tissue inflammatory signs. 

At the three-month evaluation, plaque indexes were reduced in both groups, and significantly lower (*p* < 0.05) overall VPI and mPLI values were characterized in the Test-Group compared to the Control-Group. Statistically lower mPLI was also found for Qualifying sites at day 90 in the Test-Group compared to the Control-Group.

From baseline to follow-up, overall bleeding indexes decreased in both groups. BOP varied from 75.92% to 42.23% and mBI from 1.53 to 0.43 in the Test-Group; BOP varied from 68.66% to 52.57% and mBI from 1.53 to 0.93 in the Control-Group. Intra-group examinations revealed that, from T0 to T1, reductions were greater and significantly different (*p* < 0.05) in the Test-Group compared to the Control-Group. 

At the Qualifying sites level, BOP reduction was 57.22% in the Test-Group and 42.37% in the Control-Group; mBI variations of 1.19 and 0.86 were found for the Test-Group and the Control-Group, respectively. Intra-group examinations showed statistically significant reductions from T0 to T1 for BOP and mBI in both groups. Even if the Test-Group presented greater BOP and mBI reductions, the inter-group analysis failed to find any significant differences between the groups at three-month follow-up.

At baseline, there was no statistical difference in the average PPD between groups for overall implants (Test-Group: 3.70 ± 0.83 mm; Control-Group: 3.78 ± 0.81 mm), and for Qualifying sites (Test-Group: 4.85 ± 0.99 mm; Control-Group: 5.64 ± 1.50 mm). Even if the mean PPD decreased after three months, the variations were not statistically and clinically significant for both groups. 

### 2.3. Microbiological Results

#### 2.3.1. Bacterial Count Evaluation

**Figure 1:**
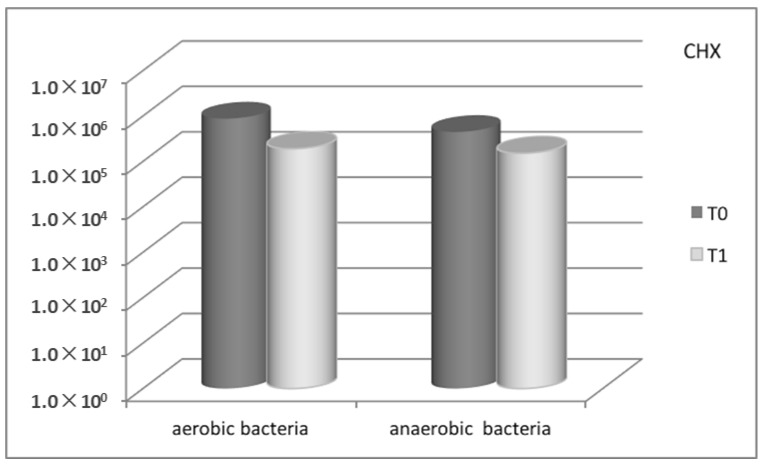
Total microbial count for Control-Group (CHX) between baseline (T0) and three-month follow-up (T1), for aerobic and anaerobic bacteria, respectively.

**Figure 2:**
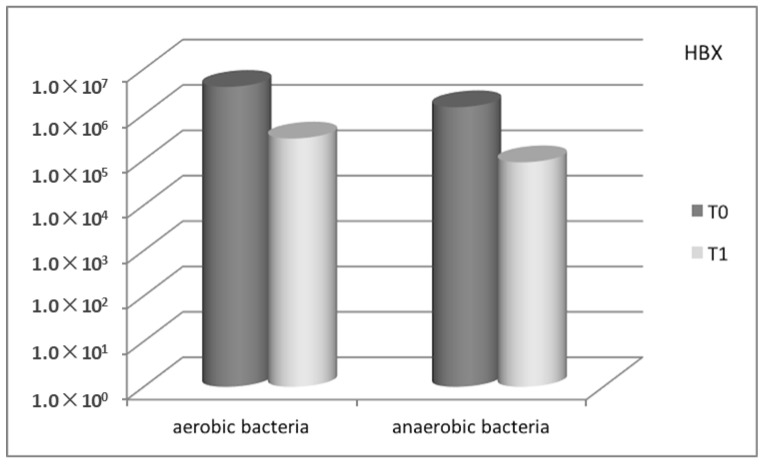
Total microbial count for Test-Group (HBX) between baseline (T0) and three-month follow-up (T1), for aerobic and anaerobic bacteria, respectively.

In the Control-Group, the comparison of microbial count, expressed as CFU/mg of plaque (colony-forming units per mg of plaque), between baseline and the three-month recall-appointment, showed a substantial decrease for all the bacteria searched, from 8.75 × 10^5^ to 1.90 × 10^5^ (mean value) for aerobic bacteria, and from 4.45 × 10^5^ to 1.51 × 10^5^ (mean value) for anaerobic bacteria (Figure 1). Also with regard to the Test-Group, a marked decrease in the microbial count was observed, from 4.07 × 10^6^ to 2.96 × 10^5^ (mean value) for aerobic bacteria, and from 1.45 × 10^6^ to 8.86 × 10^4^ (mean value) for anaerobic bacteria (Figure 2).

#### 2.3.2. Molecular Biology—Multiplex PCR (Polymerase Chain Reaction)

Concerning the data obtained with Multiplex PCR, the Control-Group showed a decrease for all three periodontopathogenic bacteria detected (*Prevotella intermedia* 12 vs. 9, *Porphyromonas gingivalis* 17 vs. 14, *Aggregatibacter actinomycetemcomitans* 13 vs 12). A decrease was observed also in the Test-Group (*P. intermedia* 12 vs. 9, *P. gingivalis* 20 vs. 14), particularly marked for *A. actinomycetemcomitans* (14 vs. 3). 

The Test-Group revealed significantly greater performances in the microbiological analysis compared to the Control-Group, exhibiting a validated effect on anaerobic bacteria, especially targeted on *A. actinomycetemcomitans*.

### 2.4. Patients’ Degree of Satisfaction

Regarding the overall degree of satisfaction with the treatment, 10% of patients were satisfied (score 3), 50% were more than satisfied (score 4), and 40% were very satisfied (score 5). In the Test-Group 10%, 40%, and 50% of patients expressed score 3, score 4, and score 5, respectively. In the Control-Group 10%, 60%, and 30% of patients expressed score 3, score 4, and score 5, respectively.

## 3. Discussion

The biofilm is a complex aggregation of microorganisms in an adhesive and protective hydrated bio-matrix (comprised of 10–30% of extracellular polymeric substances and 70% water), permeated by channels to promote the nutrient supply and the distribution of elements and signal molecules. The exopolysaccharides (EPS) of the external matrix protect bacterial cells, hinder mechanical attempts at complete biofilm removal during basic therapy, and have been demonstrated to confer increased valid resistance to disinfectants and topical or systemic antibiotics [24,25,26,27].

According to its porous structure and high water content, it has been postulated [19] that the bio-matrix could lose its integrity once exposed to the topical action of a strong desiccant agent with hygroscopic properties. As a consequence, the sub-gingival biofilm becomes particularly vulnerable to mechanical removal procedures. Recent data in the literature [19,20] has shown that hygroscopic characteristics of the sulfonated compounds present in the desiccant solution improve the effectiveness of ultrasonic debridement in the initial treatment of chronic periodontitis. 

In this prospective study, aimed to evaluate the outcomes of an adjunctive treatment protocol to manual SRP for the management of peri-implant mucositis, the same desiccant solution demonstrated greater plaque and bleeding score reductions compared to chlorhexidine gel, even if a strong statistically significant evidence was not found (probably due to the small sample size).

From a clinical point of view, our study results appeared to be in line with two previous randomized clinical trials [13,28], which experimented the topical administration of minocycline microspheres and chlorhexidine gel as adjunctive procedures to SRP in the treatment of mucositis. The topical administration of HBX showed to be effective as the administration of minocycline microspheres in decreasing the percentage of bleeding sites after probing. However, while the use of controlled-release antibiotics has to be carefully planned [29] (because of the risk of the emergence of resistant bacterial strains), HBX does not present any of these risks, and repeated administrations in a short span are possible.

BOP score offers the advantage of comparability with other studies, but it does not allow the proper determination of differences in inflammation severity [30]. mBI was hence evaluated to better understand the variations of peri-implant inflammation between groups. After three months, not only the prevalence of bleeding sites but also the severity of inflammatory signs was remarkably reduced through the administration of the desiccant solution compared to chlorhexidine gel use.

In addition, limited advantages were obtained from the use of the CHX in association with manual SRP. Other clinical trials presented similar results in the evaluation of the efficacy of chlorhexidine gel for the treatment of peri-implant mucositis. Porras et al. [12] did not show any significant benefits from the use of 0.12% chlorhexidine gel as an adjunct to SRP. Thone-Muhling et al. [31], comparing treatments of one stage full-mouth scaling with or without chlorhexidine, found significant reductions in probing depth at implant sites after eight months, with no significant differences between groups. Another study by Heitz-Mayfield et al. [18] showed that adjunctive chlorhexidine gel application did not enhance the clinical outcomes compared to mechanical debridement alone, which was effective in reducing mucositis inflammation, but not sufficient for its complete resolution. Menezes et al. [32], evaluating the use of 0.12% chlorhexidine as an adjunct to non-surgical basic therapy, observed that the use of chlorhexidine was not more effective compared to a placebo agent.

CHX presents good antibacterial activity. Nevertheless, bacterial biofilms are strongly resistant to the action of disinfectants and antibiotics, which do not counter the persistence of high bacterial loads even after treatment. Anaerobic species, in particular, are organized in a well-protected layer in deeper pockets (>4 mm) on the rough implant surface. In these cases, an easier detachment of the biofilm from the tooth/implant surface after manual treatment could, therefore, be ensured by the dehydrating action of a desiccant agent, which promotes the EPS precipitation and the entire collapse of the biofilm structure. 

The application of a desiccant solution in the present study was effective and not associated with any adverse events. These findings seem to corroborate a recently published clinical case series by Pini-Prato et al. [33], in which HBX was used to treat sites affected by peri-implant mucositis and peri-implantitis; a complete resolution of the inflammatory signs was achieved after three months without any significant adverse events. 

Even if the desiccant solution was shown to be safe and more effective than chlorhexidine gel as an adjunctive treatment to manual instrumentation, the overall clinical improvement was limited in both treated groups. In agreement with other authors [18], the results of our study seem to underline the fact that complete healing of inflamed peri-implant soft tissue is not an easily achievable goal, no matter what additional measures are associated with the manual instrumentation. 

During the clinical trial, improvements in oral hygiene conditions were observed in both groups of patients. At follow-up examinations, a statistically lower plaque accumulation was found in sites treated with the desiccant solution compared to the Control-Group. According to another study [19], which evaluated the outcomes of the desiccant solution on natural dentition, the findings deserve further studies in order to better explain our results. 

The specific role of bacteria in mucositis and peri-implantitis has been discussed in many studies; periodontal pathogens usually found in partially edentulous patients with a history of periodontal disease, also isolated from the peri-implant pockets [34] (*Aggregatibacter actinomycetemcomitans*, *Porphyromonas gingivalis*, *Prevotella intermedia*, *Tannerella forsythia*, *Treponema denticola*), represent an additional risk factor in enhancing inflammation and bone loss. Recent investigations did not show a strong association between specific microorganisms and peri-implant diseases or implant failure, but a shift from predominantly gram-positive non-motile aerobic and facultative anaerobic microorganisms towards gram-negative motile anaerobic bacteria was observed [35]. 

Both antiseptic agents examined in our study caused a decrease in the count of aerobic and anaerobic bacteria, and for all three periodontopathogens detected. Reductions in the Test-Group, in comparison with the Control-Group, were greater for all the classes of microbiota examined. Treatment with HBX resulted in a mean value reduction of more than 1 log of both aerobic and obligate anaerobic bacteria; a reduction of 78% for *A. actinomycetemcomitans* was also observed in the molecular biology investigation.

A study by Thöne-Mühling et al. [31] found no significant variation in the total bacterial load after eight months and a microbial decrease detectable after 24 hours. Heitz-Mayfield et al. [18] analyzed the proportion of the total DNA counts of orange complex species, finding >30% at one month with an unexpected decrease in the mean probing depth at three months, compared to an increase in the mean probing depth at three months if <30%. It can be assumed that changes in the proportions of specific bacterial species or complexes did not play an important role in the reduction in total bacterial counts in the treatment of peri-implant mucositis. 

The two studies by Renvert et al. [13,14] could not show any significant differences in bacterial species or group levels at any time point between the two antimicrobial agents tested. In the first one [13], the mean values of the bacterial load gradually decreased during the observation period. Furthermore, no statistical significance was evident between the two antimicrobials for any bacteria and at any time point. The second study [14] showed a similar pattern and confirmed an overall lower bacterial prevalence and level compared to cases of advanced periodontitis.

## 4. Materials and Methods 

### 4.1. Study Design

**Figure 3:**
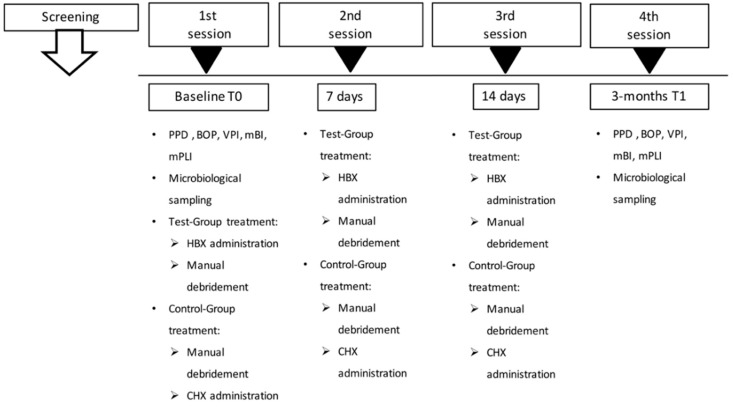
The study flow-chart.

A single-center, single-masked, parallel-groups, randomized clinical and microbiological study (Figure 3), was conducted between July and October 2018 at the Dentistry and Maxillo-facial Surgery Clinic, Department of Surgery, Dentistry, Paediatrics and Gynaecology (DIPSCOMI), University of Verona, Verona, Italy. The experimental protocol (Protocol HX-GL-ITA13, approval date 20/11/2013) was approved by the Ethical Committee of the University of Verona. The study was registered on ClinicalTrials.gov (Identifier: NCT03858959). The study followed the CONSORT Statement recommendations [36].

The study was conducted in accordance with the Ethical Principles of the 64th World Medical Association Declaration of Helsinki and was consistent with good clinical practice. All participants signed a written informed consent.

The subjects were enrolled among the individuals examined through a survey on prevalence of peri-implant infections (mucositis and peri-implantitis) among plateau-design locking-taper implants [37] with a three-year follow-up period. Twenty-three patients, aged between 37 and 71 years, met the study criteria.

Inclusion criteria comprehended the presence of at least one implant with a pocket probing depth (PPD) ≥ 4 mm, bleeding on probing (BOP), or pus on probing and no radiographically detectable bone loss (Qualifying site). Exclusion criteria were pregnant or lactating females, patients with severe systemic diseases or with uncontrolled diabetes mellitus, assumption of agents affecting the periodontal status within one month prior to the study, use of systemic antibiotics within three months prior to the study, prophylactic antibiotics requirement, peri-implant specific treatments within six months prior to the study, and allergy to sulfates and its derivatives.

Patients were randomly assigned (using a predefined computer-generated randomization scheme) to the Test-Group, which received the administration of a desiccant liquid with molecular hygroscopic properties (*HYBENX^®^ Oral Tissue decontaminant™*, HBX) before SRP, or to the Control-Group, which received the administration of a disinfectant gel after SRP (*Chlorhexidine Digluconate Corsodyl™ Dental Gel 1%*, CHX). 

A computer software (Microsoft Excel) was used to generate the random sequence, which was defined by even or odd numbers for the Test-Group or for the Control-Group, respectively. The sequence generation and proper allocation concealment were monitored by a dentist not involved in the participants’ enrolment. Opaque and sealed envelopes, each containing the secret code and bearing on the outside only a number, were opened after patients’ recruitment and informed consent signing so that the investigator involved in the enrolment and treatment could not know in advance which treatment the next person was allocated. 

### 4.2. Soft Tissues Assessment

The peri-implant soft tissues were assessed using a periodontal probe (Florida Probe; Florida Probes Company, Gainesville, FL, US), applying a force of mild intensity. The following parameters were collected at baseline (T0) and at the three-month recall appointment (T1):

- PPD, recorded in mm as the distance between the gingival margin and the base of the periodontal pocket;
- BOP, recorded as 0 (no bleeding) or 1 (bleeding) after probing for PPD;
- Modified Bleeding Index (mBI), recorded as 0, 1, 2, 3 according to Mombelli et al. [38];
- Visible Plaque Index (VPI), recorded as 0 (no plaque) or 1 (plaque) after probing for PPD;
- Modified Plaque Index (mPLI), recorded as 0, 1, 2, 3 according to Mombelli et al. [38].

Six sites on each implant were explored, three (mesial, central, distal) on the buccal side and three on the lingual/palatal side. “Qualifying site” was identified as the site with no radiographically detectable bone loss, and characterized by the deepest PPD and the presence of BOP.

Peri-implant bone levels were measured using digitally scanned intraoral radiographs, performed with a parallel technique using the Rinn centering devices [39] (Rinn XCP Posterior Aiming Ring-Yellow, Dentsply, Elgin, IL, USA).

### 4.3. Microbial Sampling and Analysis

After supra-gingival plaque removal, the deepest site was isolated with sterile cotton rolls in order to properly collect a plaque sample (1 mg ca), through two paper points inserted and left for 30 sec at the base of the periodontal pocket. 

Each collected plaque sample was divided into two Eppendorf tubes: one containing 500 μL of TE buffer (10 mM Tris-HCl, pH 8, 1 mM EDTA) for the molecular investigation by Multiplex PCR and stored at −80 °C until further processing and one containing thioglycolate medium (BD Difco) for cultural investigations. 

#### 4.3.1. Culture Investigation

The samples, before the microbiological procedures, were carefully vortex mixed for 30 sec and exposed for 30 sec to an ultrasonic cleaner (Branson mod 1210). This treatment was necessary to achieve the highest disruption and dispersion of bacteria in the sample without interfering with their culturability and avoiding DNA damaging. The plaque samples in thioglycolate medium, after appropriate dilutions (10-fold), were immediately plated on Columbia blood agar (5% sheep blood, BD Difco), in order to evaluate aerobic and facultative bacteria, and on Schaedler KV blood agar (5% Sheep Blood, kanamycin, and vancomycin BD Difco), to evaluate strict anaerobic bacteria. Plates were incubated at 37 °C for 48 hours under specific conditions; Columbia blood agar plates where incubated in a 5% CO_2_ enriched atmosphere, while Schaedler blood agar plates were placed in an anaerobic chamber (Whitley DG 250 Anaerobic Workstation, Don Whitley Scientific, Shipley, UK) with an atmosphere composed of 85% nitrogen, 10% hydrogen, and 5% CO_2_ as previously described [40]. Colonies that appeared were counted and results referred to CFU/mg. 

#### 4.3.2. DNA Extraction and Multiplex PCR Condition 

Plaque samples stored in TE buffer were used for genomic bacterial DNA extraction. Briefly, one-hundred microliters of TE samples were used for DNA extraction, using the bacterial gene DNA kit (Sigma-Aldrich), according to the manufacturer’s instructions. The extracted DNA was stored at −20 °C until the Multiplex PCR was performed. Multiplex PCR for the detection of periodontopathogenic bacteria *Porphyromonas gingivalis, Prevotella intermedia*, and *Aggregatibacter actinomycetemcomitans* was conducted using primers and conditions previously described [41].

### 4.4. Study Protocol and Treatment

At baseline (T0), subjects were randomly assigned to the Test-Group or to the Control-Group. In order to be examined for microbial sampling, peri-implant soft tissue assessment, and radiographic bone levels. The implants were consequently treated with HBX, before an SRP professional session, or CHX, after a SRP professional session. The treatment, performed by the same operator, was then repeated on days 7 and 14.

**Figure 4:**
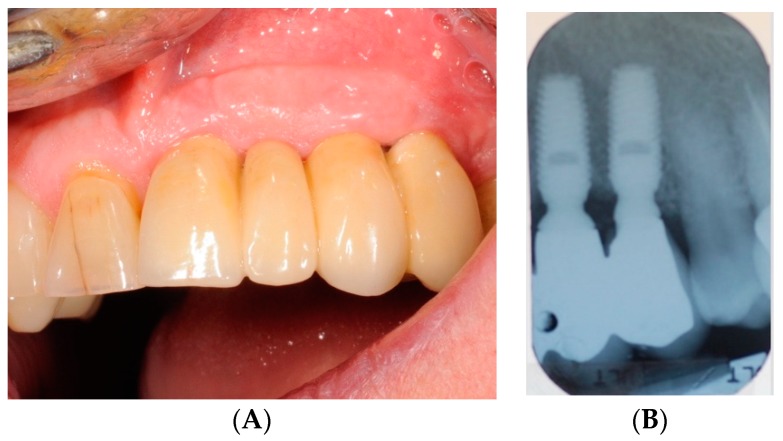
Clinical photograph (**A**) and radiograph (**B**) of a female patient at baseline (T0). The patient presented locking-taper implants in the upper jaw. One implant (site 2.4) affected by mucositis was treated with HBX (Test-Group).

**Figure 5:**
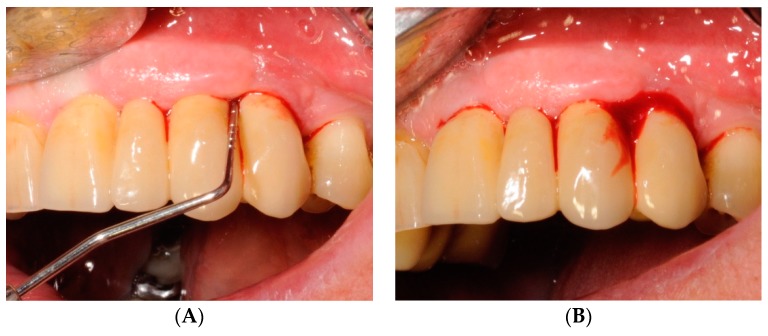
Soft tissues assessment at baseline. The implant affected by mucositis presented PPD ≥ 4 mm (**A**) and presence of BOP (**B**) and no radiographically detectable bone loss (Qualifying site).

**Figure 6:**
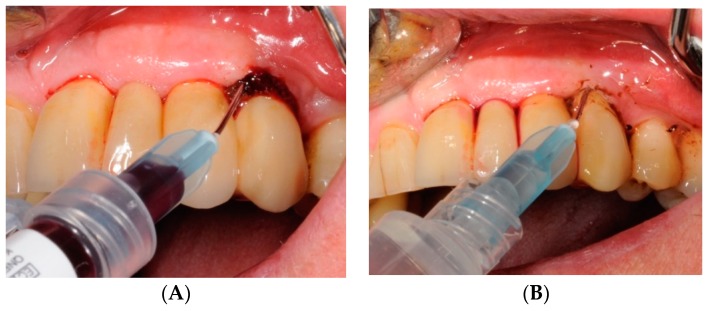
HBX application into the periodontal pocket (starting from the base) with a delivery syringe (**A**) and saline solution irrigation (**B**) after 60 sec to flush-out the product.

**Figure 7:**
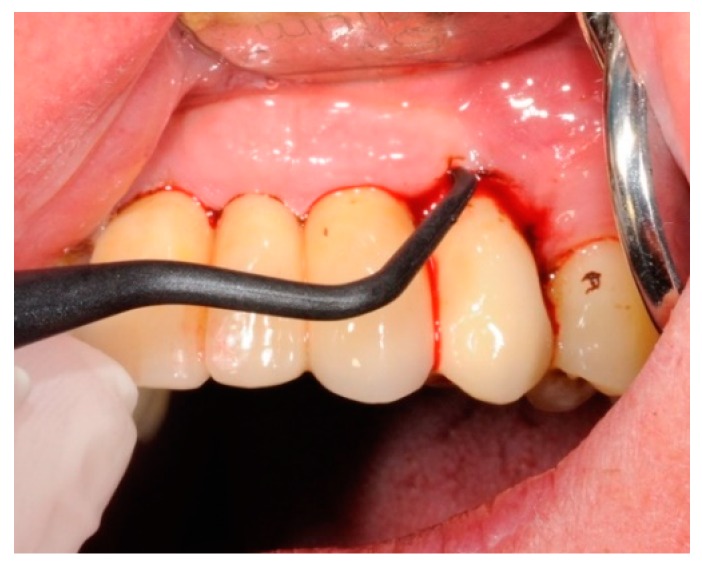
Teflon-curettes debridement to remove the deposits.

**Figure 8:**
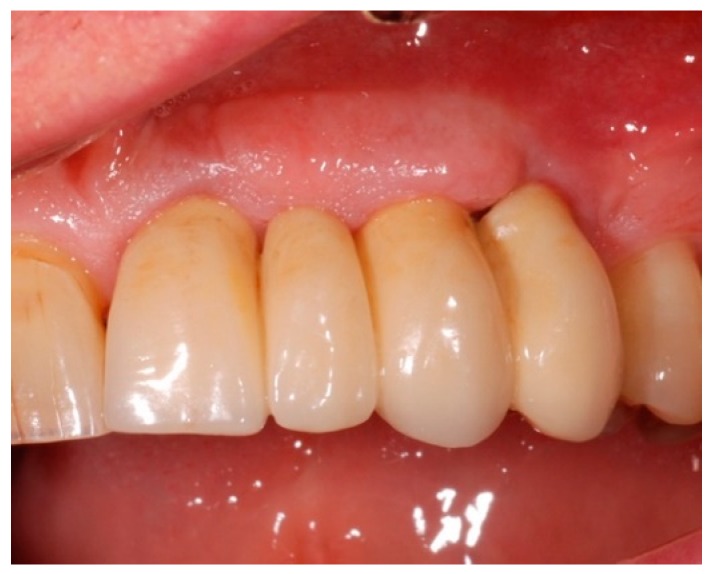
Clinical aspect at three-month follow-up (T1).

**Figure 9:**
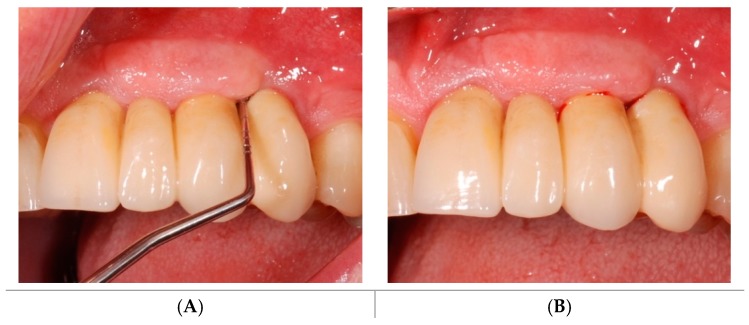
Clinical follow-up at T1: decreased PPD (**A**) and BOP (**B**) at the Qualifying site.

The HBX protocol (Figure 4, Figure 5, Figure 6, Figure 7, Figure 8 and Figure 9 show a clinical case example) consisted of [19] product application into the periodontal pocket (starting from the base) with a delivery syringe, and then saline solution irrigation after 60 sec to flush-out the product and Teflon-curettes debridement to remove the deposits.

The CHX protocol consisted of Teflon-curettes debridement to remove the deposits, associated with a first saline solution irrigation, and product application into the periodontal pocket (starting from the base) with a delivery syringe.

Data collection of soft tissue and microbiological counts were repeated at three-month follow-up (T1), together with the radiographs. 

### 4.5. Test Substances and Administration

*Chlorhexidine Digluconate Corsodyl™ Dental Gel 1%* is an antiseptic gel with cationic nature, effective against a wide range of Gram-positive and negative bacteria, favorable for plaque control and oral inflammation prevention. *HYBENX^®^ Oral Tissue decontaminant™* is a concentrated aqueous solution of sulfonated aromatics and free sulfates. Once placed onto susceptible organic material, the product instantly absorbs free and electrostatically bonded water, denaturing the molecular structure of the organic matter. Biofilm is expected to be especially sensitive to the disruptive action of HBX solution by virtue of its porous structure and high water content. In the Test-Group of this study, HBX was administered before the Teflon-curettes debridement and left in contact with the supra and sub-gingival plaque biofilm for up to 60 sec, then rinsed with water and evacuated [19].

### 4.6. Patients’ Degree of Satisfaction

At T1, patients were asked to express a personal degree of satisfaction with the completed treatment, through a numeric scale from 1 to 5 (score 1 = not at all satisfied, score 2 = partly satisfied, score 3 = satisfied, score 4 = more than satisfied, score 5 = very satisfied). 

### 4.7. Statistical Analysis

Univariate analysis was performed by assessing normality assumptions for quantitative data with the Shapiro–Wilk test; mean and standard deviations were reported for continuous data that followed a normal distribution, otherwise median and interquartile range (iqr) were reported. For the qualitative data frequencies, the proportions and 95% confidence intervals for proportions were calculated. Proportions were compared using the χ2 tests. Upon data normality and homoscedasticity check, mean comparisons were performed. Paired Student’s t-test was performed to compare a single mean in two different times over data with normal distribution, while the Mann–Whitney’s U test was carried out. Unpaired Student’s t-test was carried out to compare the means across two different groups if the data normality was found; otherwise, signed-ranked Wilcoxon test was performed. Significance level was set at 0.05, and all analyses was carried out using Stata v.13.0 for Macintosh (StataCorp, College Station, TX, USA).

## 5. Conclusions

Although both agents in association with manual debridement were demonstrated to be active in the reduction of peri-implant inflammatory signs and bacterial loads, the desiccant agent was more effective than chlorhexidine gel.

However, our findings reflect the difficulties in achieving a complete resolution of inflammation in peri-implant soft tissues. Further investigations with a larger study sample size and longer follow-ups are necessary to validate our results.

## Figures and Tables

**Table 1:** Patients and implants characteristics at baseline (T0).

Variable	Overall (*n*)	Test Group HBX (*n*)	Control Group CHX (*n*)	*p* Value
***Number of patients***	23	12	11	NS ^1^
Age ^2^	58.97 (10.09)	60.55 (6.59)	55.92 (14.45)	NS
Number of implants	52	27	25	NS
**Sex**				
Implants placed in Male	27	16	11	NS
Implants placed in Female	25	11	14	
**Smoking habit**				
Implants placed in Non-Smokers	45	23	22	NS
Implants placed in Smokers	7	4	3	
**ASA status**				
Implants placed in ASA 1 patients	32	18	14	NS
Implants placed in ASA 2 patients	20	9	11	
**Number of TPS ^3^ sessions /year**				
= 1/Year	15	7	8	NS
> 1/Year	37	20	17	
**Interproximal oral hygiene**				
No	21	12	9	NS
Yes	31	15	16	

^1^ NS: no statistically significant differences between groups. ^2^ Age is presented as mean (± standard deviation); ^3^ TPS: Periodontal supportive therapy.

**Table 2:** Soft tissues assessment for Test-Group (HBX) and Control-Group (CHX) throughout the three-month observation interval (from T0 to T1), at all six sites/implant (HBX-Group, *n* = 162; CHX-Group, *n* = 150) and at the Qualifying site/implant (HBX-Group, *n* = 27; CHX-Group, *n* = 25).

**PPD**	**All Sites/Implant (*n* = 162)**	**All Sites/Implant (*n* = 150)**	***p* Value**	**Qualifying Site/Implant (*n* = 27)**	**Qualifying Site/Implant (*n* = 25)**	***p* Value**
**Observation Interval**	**HBX Group**	**CHX Group**		**HBX Group**	**CHX Group**	
T0 (Day 0)	3.70 (0.83)	3.78 (0.81)	NS ^1^	4.85 (0.99)	5.64 (1.50)	NS
T1 (Day 90)	3.29 (0.72)	3.55 (0.83)	NS	4.37 (1.04)	5.14 (1.61)	NS
*p* value	NS	NS		NS	NS	
**mBI**	**All Sites/Implant (*n* = 162)**	**All Sites/Implant (*n* = 150)**	***p* Value**	**Qualifying Site/Implant (*n* = 27)**	**Qualifying Site/Implant (*n* = 25)**	***p* Value**
**Observation Interval**	**HBX Group**	**CHX Group**		**HBX Group**	**CHX Group**	
T0 (Day 0)	1.53 (0.55)	1.53 (0.59)	NS	2.67 (0.48)	2.43 (0.51)	NS
T1 (Day 90)	0.43 (0.34)	0.93 (0.51)	NS	1.48 (0.70)	1.57 (0.65)	NS
*p* value	<0.05 ^*^	NS		<0.05 ^*^	<0.05 ^*^	
**BOP**	**All Sites/Implant (*n* = 162)**	**All Sites/Implant (*n* = 150)**	***p* Value**	**Qualifying Site/Implant (*n* = 27)**	**Qualifying Site/Implant (*n* = 25)**	***p* Value**
**Observation Interval**	**HBX Group**	**CHX Group**		**HBX Group**	**CHX Group**	
T0 (Day 0)	75.92%	68.66%	NS	100%	100%	NS
T1 (Day 90)	42.23%	52.57%	NS	42.78%	57.63%	NS
*p* value	<0.05 ^*^	NS		<0.05 ^*^	<0.05 *	
**mPLI**	**All Sites/Implant (*n* = 162)**	**All Sites/Implant (*n* = 150)**	***p* Value**	**Qualifying Site/Implant (*n* = 27)**	**Qualifying Site/Implant (*n* = 25)**	***p* Value**
**Observation Interval**	**HBX Group**	**CHX Group**		**HBX Group**	**CHX Group**	
T0 (Day 0)	0.38 (0.46)	0.75 (0.68)	NS	0.67 (0.43)	1.21 (1.05)	NS
T1 (Day 90)	0.09 (0.20)	0.39 (0.36)	<0.05 *	0.26 (0.53)	1.14 (0.86)	<0.05 *
*p* value	<0.05 ^*^	NS		NS	NS	
**VPI**	**All Sites/Implant (*n* = 162)**	**All Sites/Implant (*n* = 150)**	***p* Value**	**Qualifying Site/Implant (*n* = 27)**	**Qualifying Site/Implant (*n* = 25)**	***p* Value**
**Observation Interval**	**HBX Group**	**CHX Group**		**HBX Group**	**CHX Group**	
T0 (Day 0)	31.48%	54.76%	NS	44.54%	29.01%	NS
T1 (Day 90)	8.64%	26.19%	<0.05 *	7.43%	10.74%	NS
*p* value	<0.05 ^*^	<0.05 ^*^		<0.05 ^*^	NS	

Values are presented as mean (± standard deviation); ^1^ NS: no statistically significant differences between groups; * Statistically significant differences between groups/observation times. PPD: pocket probing depth (in mm); mBI: modified bleeding index; BOP: bleeding on probing; mPLI: modified plaque index; VPI: visible plaque index; T0 (Day 0): baseline; T1 (Day 90): three-month follow-up.

## Supplementary Materials

The following are available online at https://www.mdpi.com/2079-6382/8/2/82/s1, Table S1: Consort 2010 checklist of information to include when reporting a randomised trial.
